# Supplementary material for: A clarifying perspective on bacterial pseudo-receiver domains
Source: J Bacteriol. 2025 Oct 8;207(11):e00261-25. doi: 10.1128/jb.00261-25 (PMC12632262; doi:10.1128/jb.00261-25)
Supplement: Supplemental material — s and methods; Descriptions of Datasets S1 to S5, including links to Datasets S1 and S2; Tables S1 to S3. [file jb.00261-25-s0001.pdf]

For submission to the *Journal of Bacteriology*
6/24/25

SUPPLEMENTAL MATERIAL

A Clarifying Perspective on Bacterial Pseudo-Receiver Domains

Robert B. Bourret[1#], Emily N. Kennedy[1], Rita Tamayo[1], Clay A. Foster[2]

[1]Department of Microbiology and Immunology, University of North Carolina, Chapel Hill, North Carolina, USA

[2]Department of Pediatrics, Section Hematology/Oncology, University of Oklahoma Health Sciences Center, Oklahoma City, Oklahoma, USA

Running Head: Pseudo-Receiver Domains

[#]Address correspondence to Robert B. Bourret, bourret@med.unc.edu

MATERIALS AND METHODS

Obtaining sequence sets. UniProtKB (1) entries with corresponding InterPro domains were downloaded from the InterPro database (2) (August 14th, 2023). Entries were filtered to retain only proteins with either a detectible receiver (IPR001789/PF00072; note that this includes PsR domains), Hpt (IPR008207/PF01627), HisKA (IPR003661/PF00512) or HisKA_3 (IPR011712 /PF07730) domain. A list of representative proteomes (3) (specifically RP75) was obtained from the Protein Information Resource database (4). UniProtKB entries described above were further filtered to retain proteins from representative proteomes only (numerous proteomes contained multiple distinct proteins with full receiver domains). Full-length sequences were obtained for the retained entries and initial domain sizes were estimated using the existing InterPro domain boundaries. Taxonomic lineage information was incorporated based on the NCBI Taxonomy Database (5, 6). To minimize taxonomic bias, sequences from genera containing multiple distinct species were subsetted to retain only a single randomly selected representative species. AlphaFold models were used for manual validation and for secondary structure identification. Sequences lacking a corresponding model in the AlphaFold database (v.4) (7-9) were excluded. Finally, sequence sets were filtered to exclude non-bacterial proteins.

Processing and distinguishing receiver and PsR domain sequences. A small percentage of bacterial receiver domain sequences exhibited abnormally short domain sizes (< 60 residues), likely indicating incomplete sequencing data, and were excluded. The full-length, single-domain receiver protein *E. coli* CheY was added to the beginning

of the set as a reference sequence. To alleviate domain prediction ambiguity at the termini of the sequences, predicted receiver domain boundaries were expanded by 20 residues on both the N- and C-termini (or as much as possible if already near the true terminus of the protein) for the retained sequence set. If the resulting expanded domain length was still < 110 residues (the estimated average receiver domain size), then additional positions were added symmetrically (where possible) until the 110-residue threshold was reached. Sequences shorter than 110 residues after this extension process were excluded, and highly redundant expanded sequences were filtered out using CD-HIT (v.4.8.1; cutoff of 90%) (10). AlphaFold models for the remaining domain sequences were downloaded and secondary structure predictions were extracted from the resulting .cif/.pdb files (based on a three-state model) with the *mkdssp* program (11, 12). More exact domain boundaries were established by identifying subsequences capturing the canonical $(\beta\alpha)_5$ secondary structure topology. If multiple subsequence orientations (“matches”) were found, the “correct” one was determined by maximizing the number of catalytic landmarks detected. The secondary structure predictions were used to search for these catalytic landmark residues based on the following criteria: a metal-binding acidic residue pair (DD, ED, EE, or DE; termed DD) near the C-terminus of β1, a phosphorylatable Asp residue (termed D) near the C-terminus of β3, a Ser or Thr (termed T) near the C-terminus of β4, and a Lys (termed K) near the C-terminus of β5. If no ideal $(\beta\alpha)_5$ match was identified for a given sequence, isolated or likely erroneous secondary structure assignments (e.g., two-residue long helices or strands, short coils breaking up otherwise contiguous structures, etc.) were corrected for and the process was repeated. Domains lacking $(\beta\alpha)_5$ topology even after these corrections

were excluded from the data set. Extraneous flanking residues outside of the predicted central $(\beta\alpha)_5$ topology of a canonical receiver domain were trimmed from each sequence. Additionally, some receiver domains feature a highly extended α5 helix (often connecting to an output domain). Because this might throw off alignment of the core conserved domain structure, these extended helices were truncated to a maximum of 17 residues (the overall average length of α5 in the sequence set). A given sequence was classified as a PsR if it lacked any of the four catalytic landmarks mentioned above. Of note, many PsRs encode a Glu in place of (or sometimes in addition to) the phosphorylatable Asp. In this scenario, the presence of the D landmark was confirmed if an Asp residue was closer to the C-terminus of β3 than any additional acidic residue. The above protocol resulted in 9,153 PsR sequences (out of a total of 152,269 sequences) or ~6% of our non-redundant representative bacterial sequence set. WITCH (WeIghTed Consensus Hmm alignment; witch-msa v.1.0.7) (13) was utilized to generate a sequence alignment of all retained sequences using default parameters and then split into separate PsR (**Dataset S1**) and receiver domain (**Dataset S2**) sub-alignments. The two sub-alignments were analyzed to determine pairwise mutual information scores and amino acid abundances at key positions in the domain relative to the *E. coli* CheY reference sequence. To improve interpretability, gaps in the alignments were deleted in relation to the CheY sequence in both sub-alignments.

Amino acid composition and mutual information analysis. The bacterial PsR and true receiver domain multiple sequence alignments obtained as described above were submitted to the MISTIC server for corrected mutual information analysis (14). Amino acid frequency tables (**Datasets S3 and S4**) were manually calculated using the

seqinr package (v.4.2-36) (15) and converted to percentages. A difference matrix was created to demonstrate shifts in amino acid residue frequencies by subtracting the frequencies detected in the PsR alignment from those found in the true receiver domain alignment. Therefore, positive values represent higher frequency in bona fide receiver domains, whereas negative values represent higher frequency in PsRs.

DATASETS

Dataset S1. Multiple sequence alignment of 9,153 bacterial PsR sequences in FASTA format

E. coli CheY added as a reference. Available at https://doi.org/10.5281/zenodo.15726377.

Dataset S2. Multiple sequence alignment of 143,116 bacterial true receiver domain sequences in FASTA format

Already includes *E. coli* CheY. Available at https://doi.org/10.5281/zenodo.15726377.

Dataset S3. Amino acid frequency by position in bacterial PsR sequences.

Calculated from Dataset S1 as described in Materials and Methods. Available as a separate file.

Dataset S4. Amino acid frequency by position in bacterial true receiver domain sequences.

Calculated from Dataset S2 as described in Materials and Methods. Available as a separate file.

Dataset S5. Amino acid co-abundance calculator for bacterial PsRs.

Based on Dataset S1. Calculates the number of amino acids at any PsR position *j* given a specific amino acid at position *i*. The multiple sequence alignment has 120 positions corresponding to positions 8-127 of the representative receiver domain *E. coli* CheY. Note that positions are not specified as 8 through 127, but rather as D ± n, where D corresponds to position 57 (the conserved phosphorylation site of receiver domains). Similarly, position DD1 is D-45, DD2 is D-44, T is D+30, and K is D+52. Inclusion of *E.*

coli CheY in calculations for simplicity introduces a negligible ~0.01% error. Available as a separate file.

TABLES

Table S1. Amino acid frequencies at key receiver and PsR domain positions

Position	Amino Acid	Amino Acid Composition (%)[a] Rec	PsR	Function
A. Active Site Positions				
DD1	Asp	62	58	Indirect metal binding
	Glu	38	24	
DD2	Asp	100	28	Direct metal binding
	Asn	0	19	
	Ser	0	9	
	Pro	0	8	
	Ala	0	7	
	Gly	0	7	
D	Asp	100	63	Phosphorylation site
	Glu	0	12	
T	Thr	69	38	Coordinate phosphoryl group
	Ser	31	32	
	Ala	0	11	
K	Lys	100	64	Coordinate phosphoryl group
	Arg	0	11	
B. Catalytic Variable Positions				
DD+1	Glu	30	11	Metal binding?
	Asp	29	32	
	Asn	11	14	
	His	10	7	
	Ser	9	12	
D+2	Met	20	4	Interaction with attacking or leaving group
	Arg	15	9	
	Asn	12	9	
	Gln	10	7	
T+1	Ala	52	29	Access to phosphorylation site
	Gly	22	14	
	Ser	10	14	
	Thr	8	5	
T+2	Tyr	17	5	Interaction with attacking or leaving group
	Arg	14	8	
	His	11	6	
	Lys	10	4	
	Phe	10	4	

C. Structural Positions

D+4	Pro	88	53	Hairpin turn in β3α3 loop
D+8	Gly	97	73	N-terminus of α3 helix

D. Allosteric Pathway – D to α4β5α5 Surface

D+1	Ile	34	13
	Leu	25	25
	Val	22	14
T-2	Met	23	15
	Phe	18	11
	Val	18	21
	Ile	16	14
	Leu	13	23
	Ala	11	9
T	Thr	69	38
	Ser	31	32
	Ala	0	11
T+2	Tyr	17	5
	Arg	14	8
	His	11	6
	Lys	10	4
	Phe	10	4
K-3	Tyr	62	43
	Phe	24	22
	Val	4	11

E. Allosteric Pathway – D to α1α5 Surface

DD+4	Ile	23	12
	Val	17	13
	Leu	15	11
	Asn	12	8
DD+8	Leu	48	46
	Ile	17	17
	Val	13	9
DD+11	Leu	22	25
	Ile	12	10
	Ala	11	10
DD+12	Leu	78	77
T-3	Ile	48	41
	Leu	24	23
	Val	22	24

T-1	Leu	50	34
	Val	16	9
	Ile	14	19
K+1	Pro	80	71
K+2	Phe	40	20
	Val	15	20
	Ile	12	13
	Leu	6	16
	Ala	6	6
K+4	Pro	23	27
	Leu	12	8
	Ala	8	11
K+7	Leu	72	69
	Ile	8	8
	Val	8	9

[a] From Datasets S3 and S4.

Table S2. PsR domain networks inferred from mutual information analysis

Position	*E. coli* CheY Position	Location[a]	Inter-network interactions (position)	Comments
A. Network 1 (orange in Fig. 3A)				
Core network[b]				
DD+1	14	N-terminal α1		
DD+3	16	N-terminal α1		
DD+4	17	α1		
DD+7	20	α1	6 core (105)	
K	109	β5α5 loop		
Extended network[b]				
DD1	12	β1α1 loop		
DD+11	24	α1		
K+4	113	N-terminal α5		
B. Network 2 (yellow in Fig. 3A)				
Core network				
DD+5	18	α1		In top 10 highest PsR MI[c] scores
DD+11	22	α1		
D-22	35	β2	6 core (34; 36)	In top 10 highest PsR MI scores
D-20	37	β2α2 loop	4 core (41)	
Extended network				
DD+13	26	C-terminal α1		
C. Network 3 (blue in Fig. 3A)				
Core network				
DD+15	28	C-terminal α1	4 core (83); 6 core (58; 59)	In top 10 highest PsR cMI[d] scores
DD+18	31	α1β2 loop		
K+8	117	α5		
Extended network				
DD-5	8	N-terminal β1		

D. Network 4 (red in Fig. 3B)

<u>Core network</u>

DD2	13	β1α1 loop	6 core (58; 59)	
D-19	38	N-terminal α2		
D-18	39	N-terminal α2	6 core (11)	In top 10 highest PsR MI/cMI scores
D-16	41	α2	2 core (37)	
D-14	43	α2		
D-1	56	C-terminal β3		
D+3	60	β3α3 loop		In top 10 highest PsR MI/cMI scores
D+4	61	β3α3 loop		
D+5	62	β3α3 loop		
D+6	63	β3α3 loop		In top 10 highest PsR MI/cMI scores
D+7	64	N-terminal α3		
D+8	65	N-terminal α3		
D+11	68	α3		
D+12	69	α3	6 core (73; 85)	
T-4	83	N-terminal β4	3 core (28); 6 core (58)	

<u>Extended network</u>

D-17	40	N-terminal α2	
D-3	54	β3	6 core (81)
D	57	β3α3 loop	
D+10	67	α3	6 extended (70)
D+14	71	α3	

E. Network 5 (magenta in Fig. 3C)

<u>Core network</u>

D-11	46	α2	
T-15	72	α3	
T-12	75	C-terminal α3	
T-11	76	α3β4 loop	
K-3	106	β5	6 core (99)
K-1	108	C-terminal β5	

K+9	118	α5	6 core (115; 119)	
K+16	125	α5	6 core (104)	

<u>Extended network</u>

T-8	79	α3β4 loop		

F. Network 6 (cyan in Fig. 3D)

<u>Core network</u>

DD-4	9	β1		
DD-2	11	C-terminal β1	4 core (39)	In top 10 highest PsR MI scores
D-25	32	α1β2 loop		In top 10 highest PsR MI scores
D-23	34	β2	2 core (35)	In top 10 highest PsR MI/cMI scores
D-21	36	C-terminal β2	2 core (35)	In top 10 highest PsR MI scores
D-15	42	α2		In top 10 highest PsR MI scores
D-12	45	α2		In top 10 highest PsR MI scores
D+1	58	β3α3 loop	3 core (28); 4 core (13; 83)	In top 10 highest PsR cMI scores
D+2	59	β3α3 loop	3 core (28); 4 core (13)	
D+16	73	C-terminal α3	4 core (69)	In top 10 highest PsR MI scores
T-6	81	α3β4 loop	4 core (54)	
T-5	82	α3β4 loop		
T-2	85	β4	4 core (69)	
T-1	86	β4		
T+2	89	β4α4 loop		
T+7	94	α4		
K-10	99	α4	5 core (106)	
K-7	102	α4β5 loop		
K-6	103	α4β5 loop		
K-5	104	α4β5 loop	5 core (125)	In top 10 highest PsR MI/cMI scores
K-4	105	N-terminal β5	1 core (20)	In top 10 highest PsR MI/cMI scores
K+1	110	β5α5 loop		In top 10 highest PsR MI/cMI scores
K+2	111	β5α5 loop		In top 10 highest PsR MI scores
K+6	115	α5	5 core (118)	
K+10	119	α5	5 core (118)	In top 10 highest PsR MI/cMI scores

K+13	122	α5		
K+14	123	α5		In top 10 highest PsR MI scores
K+15	124	α5		
<u>Extended network</u>				
D-4	53	N-terminal β3		
D+13	70	α3	4 extended (67)	
T-3	84	β4		
T+4	91	N-terminal α4		
T+5	92	N-terminal α4		
T+8	95	α4		
T+9	96	α4		
T+10	97	α4		
T+11	98	α4		
K-8	101	C-terminal α4		

[a] Based on the structure of *E. coli* CheY (PDB ID 1fqw). N- or C-terminal indicates within three residues of end of α helix or within one residue of end of β strand.

[b] Core networks are composed of isolated residue groups intra-connected by mutual information scores in the top 0 to 1%. Extended networks are composed of additional residues connected to existing core groups by mutual information scores in the top 1 to 2%. Only first-order neighbors are considered as part of additional networks via extended interactions. Extended networks were identified by merging core isolated networks with mutual information interactions from the top 1 to 2% within Cytoscape (16) and applying a best neighbor filter.

[c] Mutual Information as calculated by MISTIC (14).

[d] Cumulative MI = sum of all statistically significant (≥ 6.5) MI scores involving the indicated residue. High cMI is often associated with functional and/or catalytic residues (14).

Table S3. Experimentally determined[a] structures of PsR domains[b]

PBD ID	Species	Protein[c]	"DD D T K"[d]	Dimer?[e]	Comments	Source
PF00072 Response regulator receiver domain (2M InterPro entries)						
3luf	*Aeromonas salmonicida*	A4SNL2	DD D S Q	α4β5α5	1/2 length, Rec and PsR domains	NYSGXRC
3mf4	*Aeromonas salmonicida*	A4SNL2	DD D S Q	α4β5α5	1/2 length, Rec and PsR domains	NYSGXRC
4dad	*Burkholderia pseudomallei*	Q63I75	ED D T W	α4β5	α2 kinked, no γ turn	JCSG
4dn6	*Burkholderia pseudomallei*	Q2T2Y8	ED D S W	α4β5	α2 kinked, no γ turn	JCSG
1w25	*Caulobacter crescentus*	PleD	DD N V R	β5α5	Full length PleD•c-di-GMP, Rec (D1) & PsR (D2) domains. "Dimer" interface between Rec and PsR of same chain	(17)
2v0n	*Caulobacter crescentus*	PleD	DD N V R	α4β5α5	Full length PleD•BeF_3^-•c-di-GMP•GTPαS, Rec (D1)& PsR (D2) domains. "Dimer" interface between Rec and PsR of same chain	(18)
2wb4	*Caulobacter crescentus*	PleD	DD N V R	α4β5α5	Full length PleD•BeF_3^-•c-di-GMP, Rec (D1) & PsR (D2) domains. "Dimer" interface between Rec and PsR of same chain	Wassman *et al.*, unpublished
6qrl	*Caulobacter crescentus*	ShkA	AS D T K	No	ShkA PsR•c-di-GMP, α2 short, no α3, α4 bent, no γ turn, c-di-GMP binds to α4β5α5, ShkA is a HHK	(19)
6qrj	*Caulobacter crescentus*	ShkA	AS D T K	No	Full length ShkA•AMPPNP, PsR and Rec domains, α2 short, no α3, α4 bent, no γ turn, ShkA is a HHK	(19)
2qzj	*Clostridiodes difficile*	CmrT	DG E T K	α4β5α5		NYSGXRC
3lua	*Clostridium thermocellum*	A3DK02	DY D T K	No	A3DK02 is a HHK	NYSGXRC
2zay	*Desulfuromonas acetoxidans*	Q1JZD9	DT E S K	α4β5α5		NYSGXRC

2pln	*Helicobacter pylori*	HP1043	EK S S K	α4β5α5	No γ turn	(20)
2hqo	*Helicobacter pylori*	HP1043	EK S S K	α4β5α5	NMR, no γ turn,	(20)
2hqr	*Helicobacter pylori*	HP1043	EK S S K	α4β5α5	NMR, full length, no γ turn	(20)
2gkg	*Myxococcus xanthus*	FrzS	ES A G K	No		(21)
2i6f	*Myxococcus xanthus*	FrzS	ES A G K	No		(21)
2nt3	*Myxococcus xanthus*	FrzS	ES A G K	No	Y102A mutant	(21)
2nt4	*Myxococcus xanthus*	FrzS	ES A G K	No	H92F mutant	(21)
3nhm	*Myxococcus xanthus*	Q1CZZ7	EN D S K	No	No α4	NYSGXRC
3n53	*Pelobacter carbinolicus*	Q3A6W4	DQ D S K	No	No α4	NYSGXRC
3kto	*Pseudoalteromonas atlantica*	A0A9J9BB43	DH E A K	No		NYSGXRC
3hv2	*Pseudomonas fluorescens*	Q4K707	DS A T K	α4β5		NYSGXRC
2j48	*Synechococcus elongatus*	CikA	EE A L K	No	NMR, α4 and β5 short, CikA is a HHK, Quinones bind to α1β2	(22)
5jyu	*Thermosynechococcus elongatus*	CikA	ED E S L	No	NMR, α2 short, no α4, CikA is a HHK	(23)
5jyv	*Thermosynechococcus elongatus*	CikA	ED E S L	No	NMR, CikA PsR•KaiB, α2 short, no α4, binds to KaiB on α1β2α2 surface, CikA is a HHK	(23)
4lzl	*Streptococcus pneumoniae*	RitR	EK N D K	No	α4 kinked	(24)
5u8m	*Streptococcus pneumoniae*	RitR	EK N D K	Cys128	Full length, α4 kinked	(25)
5u8k	*Streptococcus pneumoniae*	RitR	EK N D K	No	Full length C128S mutant, α4 kinked	(25)
5vfa	*Streptococcus pneumoniae*	RitR	EK N D K	No	Full length C128D mutant, α4 kinked	(25)

PF06490 Flagellar regulatory protein FleQ (3K InterPro entries)

4wxm	*Pseudomonas aeruginosa*	FleQ	DD G G M	α1	α2 short, α4 short	(26)
8p53	*Pseudomonas aeruginosa*	FleQ	DD G G M	α1	Cryo-EM, full length FleQ•FleN, α2 short, α4 short	(27)
8pb9	*Pseudomonas aeruginosa*	FleQ	DD G G M	α1	Cryo-EM, 2/3 FleQ (PsR-AAA+)•c-di-GMP•FleN, α2 short, α4 short	(27)
8h5v	*Vibrio cholerae*	FlrA	ED G N F	No	α2 short, no α4	(28)
<u>*PF21155 VpsT-like, receiver domain (2K InterPro entries)*</u>						
3kln	*Vibrio cholerae*	VpsT	SD D C D	α1	Full length, α2 kinked, α4 short	(29)
3klo	*Vibrio cholerae*	VpsT	SD D C D	α6	Full length VpsT•di-c-GMP, α2 kinked, α4 short	(29)
5xp0	*Escherichia coli*	CsgD	TK D T M	α1	No α2, α4 short	(30)
<u>*PF21194 TadZ-like, receiver domain (241 InterPro entries)*</u>						
3fkq	*Eubacterium rectale*	TadZ	DK E T K	NA	Full length TadZ•ATP, α3 is a short 3_{10} helix, no α4	(31)
<u>*PF21695 Transcription regulator GlnR, N-terminal domain (5K InterPro entries)*</u>						
4o1h	*Amycolatopsis mediterranei*	GlnR	TA D V L	α4β5α5	α1 and α4 kinked, α2 short, no γ turn	(32)
4o1i	*Mycobacterium tuberculosis*	GlnR	TS D V L	α4β5α5	α1 and α4 kinked, α2 short, no γ turn	(32)
8hih	*Mycobacterium tuberculosis*	GlnR	TS D V L	No	Cryo-EM, GlnR•RNA polymerase•DNA complex, α1 and α4 kinked, α2 short	(33)
<u>*PF21714 Circadian clock protein KaiA, N-terminal domain (508 InterPro entries)*</u>						
1m2e/ 1m2f	*Synechococcus elongatus*	KaiA	ES V G I	No	NMR, no α4, no γ turn Family of 25 NMR structures	(34)
1r8j	*Synechococcus elongatus*	KaiA	ES V G I	NA	Full length, no α4, no γ turn	(35)

4g86	*Synechococcus elongatus*	KaiA	ES V G I	NA	Full length KaiA•DBMIB, no α4, no γ turn DBMIB quinone binds near the β3α3 loop/ N-terminal end of α3	(36)
5c5e	*Synechococcus elongatus*	KaiA	ES V G I	NA	Full length KaiA•KaiC peptide, no α4, no γ turn	(37)
PF22368 Response regulator protein ChxR, N-terminal domain (19 InterPro entries)						
3q7r	*Chlamydia trachomatis*	ChxR	EH E D R	α4β5α5	No α2 or α3, no γ turn	(38)
3q7s	*Chlamydia trachomatis*	ChxR	EH E D R	α4β5α5	No α2 or α3, no γ turn	(38)
3q7t	*Chlamydia trachomatis*	ChxR	EH E D R	α4β5α5	No α2 or α3, no γ turn	(38)
No Pfam domain identified						
3snk	*Mesorhizobium loti*	Q989D4	SS D S K	No	α2 short, no γ turn	JCSG

[a]X-ray unless otherwise noted.

[b]Structure of PsR domain only unless otherwise noted

[c]UNIPROT identifier if protein unnamed

[d]PsR residues corresponding to receiver domain DD D T K conserved residues.

[e]PsR dimer interface indicated if dimer observed. NA (not applicable) indicates dimer but not via PsR domain.

Structures excluded from Table S3

- *Pseudomonas aeruginosa* AmiR (PBD ID 1qo0) was identified as containing a PsR domain in 1999 (39). However, this identification has not stood the test of time. The order of secondary structural elements encoded in receiver domain primary sequence is β1-α1-β2-α2-β3-α3-β4-α4-β5-α5 (40), whereas in AmiR the order is α1-β1-α2-β2-β3-α3-

β4-α4-β5-α5 (PBD ID 1qo0). Pfam classifies the domain as PF21332 AmiR, N-terminal, not PF00072 or any of the minor Pfam groupings included in Table S3.

- *Alkalihalobacillus halodurans* BH3024 (PBD ID 2b4a) was identified as a PsR in (24), which is something of a judgement call. BH3024 clearly contains the conserved DD, T, and K residues. Therefore, categorization of BH3024 depends on which residue is identified as corresponding to the D position. The BH3024 residue at the C-terminal end of β3 is a Ser, which presumably is the basis of classification as a PsR. However, the next residue is an Asp, and there are many receiver domains (including the well characterized model receiver domain *E. coli* CheY) in which the phosphorylation site Asp is immediately preceded by a Ser. For the purposes of this review, we regard BH3024 to be a receiver, not a PsR. A definitive experimental test to distinguish between PsR and receiver would be to determine if BH3024 can be phosphorylated on the Asp.

REFERENCES

1. UniProt C. 2023. UniProt: the Universal Protein Knowledgebase in 2023. Nucleic Acids Res 51:D523-D531.
2. Paysan-Lafosse T, Blum M, Chuguransky S, Grego T, Pinto BL, Salazar GA, Bileschi ML, Bork P, Bridge A, Colwell L, Gough J, Haft DH, Letunic I, Marchler-Bauer A, Mi H, Natale DA, Orengo CA, Pandurangan AP, Rivoire C, Sigrist CJA, Sillitoe I, Thanki N, Thomas PD, Tosatto SCE, Wu CH, Bateman A. 2023. InterPro in 2022. Nucleic Acids Res 51:D418-D427.
3. Chen C, Natale DA, Finn RD, Huang H, Zhang J, Wu CH, Mazumder R. 2011. Representative proteomes: a stable, scalable and unbiased proteome set for sequence analysis and functional annotation. PLoS One 6:e18910.
4. Wu CH, Yeh LS, Huang H, Arminski L, Castro-Alvear J, Chen Y, Hu Z, Kourtesis P, Ledley RS, Suzek BE, Vinayaka CR, Zhang J, Barker WC. 2003. The Protein Information Resource. Nucleic Acids Res 31:345-7.
5. Sayers EW, Cavanaugh M, Clark K, Ostell J, Pruitt KD, Karsch-Mizrachi I. 2019. GenBank. Nucleic Acids Res 47:D94-D99.
6. Schoch CL, Ciufo S, Domrachev M, Hotton CL, Kannan S, Khovanskaya R, Leipe D, McVeigh R, O'Neill K, Robbertse B, Sharma S, Soussov V, Sullivan JP, Sun L, Turner S, Karsch-Mizrachi I. 2020. NCBI Taxonomy: a comprehensive update on curation, resources and tools. Database (Oxford) 2020.
7. Jumper J, Evans R, Pritzel A, Green T, Figurnov M, Ronneberger O, Tunyasuvunakool K, Bates R, Zidek A, Potapenko A, Bridgland A, Meyer C, Kohl SAA, Ballard AJ, Cowie A, Romera-Paredes B, Nikolov S, Jain R, Adler J, Back T,

Petersen S, Reiman D, Clancy E, Zielinski M, Steinegger M, Pacholska M, Berghammer T, Bodenstein S, Silver D, Vinyals O, Senior AW, Kavukcuoglu K, Kohli P, Hassabis D. 2021. Highly accurate protein structure prediction with AlphaFold. Nature 596:583-589.

8. Varadi M, Anyango S, Deshpande M, Nair S, Natassia C, Yordanova G, Yuan D, Stroe O, Wood G, Laydon A, Zidek A, Green T, Tunyasuvunakool K, Petersen S, Jumper J, Clancy E, Green R, Vora A, Lutfi M, Figurnov M, Cowie A, Hobbs N, Kohli P, Kleywegt G, Birney E, Hassabis D, Velankar S. 2022. AlphaFold Protein Structure Database: massively expanding the structural coverage of protein-sequence space with high-accuracy models. Nucleic Acids Res 50:D439-D444.

9. Varadi M, Bertoni D, Magana P, Paramval U, Pidruchna I, Radhakrishnan M, Tsenkov M, Nair S, Mirdita M, Yeo J, Kovalevskiy O, Tunyasuvunakool K, Laydon A, Zidek A, Tomlinson H, Hariharan D, Abrahamson J, Green T, Jumper J, Birney E, Steinegger M, Hassabis D, Velankar S. 2024. AlphaFold Protein Structure Database in 2024: providing structure coverage for over 214 million protein sequences. Nucleic Acids Res 52:D368-D375.

10. Fu L, Niu B, Zhu Z, Wu S, Li W. 2012. CD-HIT: accelerated for clustering the next-generation sequencing data. Bioinformatics 28:3150-2.

11. Kabsch W, Sander C. 1983. Dictionary of protein secondary structure: pattern recognition of hydrogen-bonded and geometrical features. Biopolymers 22:2577-637.

12. Touw WG, Baakman C, Black J, te Beek TA, Krieger E, Joosten RP, Vriend G. 2015. A series of PDB-related databanks for everyday needs. Nucleic Acids Res 43:D364-8.

13. Shen C, Park M, Warnow T. 2022. WITCH: Improved multiple sequence alignment through weighted consensus hidden Markov model alignment. J Comput Biol 29:782-801.

14. Simonetti FL, Teppa E, Chernomoretz A, Nielsen M, Marino Buslje C. 2013. MISTIC: Mutual information server to infer coevolution. Nucleic Acids Res 41:W8-14.

15. Gouy M, Milleret F, Mugnier C, Jacobzone M, Gautier C. 1984. ACNUC: a nucleic acid sequence data base and analysis system. Nucleic Acids Res 12:121-7.

16. Shannon P, Markiel A, Ozier O, Baliga NS, Wang JT, Ramage D, Amin N, Schwikowski B, Ideker T. 2003. Cytoscape: a software environment for integrated models of biomolecular interaction networks. Genome Res 13:2498-504.

17. Chan C, Paul R, Samoray D, Amiot NC, Giese B, Jenal U, Schirmer T. 2004. Structural basis of activity and allosteric control of diguanylate cyclase. Proc Natl Acad Sci U S A 101:17084-9.

18. Wassmann P, Chan C, Paul R, Beck A, Heerklotz H, Jenal U, Schirmer T. 2007. Structure of BeF_3^--modified response regulator PleD: implications for diguanylate cyclase activation, catalysis, and feedback inhibition. Structure 15:915-27.

19. Dubey BN, Agustoni E, Bohm R, Kaczmarczyk A, Mangia F, von Arx C, Jenal U, Hiller S, Plaza-Menacho I, Schirmer T. 2020. Hybrid histidine kinase activation by

cyclic di-GMP-mediated domain liberation. Proc Natl Acad Sci U S A 117:1000-1008.

20. Hong E, Lee HM, Ko H, Kim DU, Jeon BY, Jung J, Shin J, Lee SA, Kim Y, Jeon YH, Cheong C, Cho HS, Lee W. 2007. Structure of an atypical orphan response regulator protein supports a new phosphorylation-independent regulatory mechanism. J Biol Chem 282:20667-75.

21. Fraser JS, Merlie JP, Jr., Echols N, Weisfield SR, Mignot T, Wemmer DE, Zusman DR, Alber T. 2007. An atypical receiver domain controls the dynamic polar localization of the *Myxococcus xanthus* social motility protein FrzS. Mol Microbiol 65:319-32.

22. Gao T, Zhang X, Ivleva NB, Golden SS, LiWang A. 2007. NMR structure of the pseudo-receiver domain of CikA. Protein Sci 16:465-75.

23. Tseng R, Goularte NF, Chavan A, Luu J, Cohen SE, Chang YG, Heisler J, Li S, Michael AK, Tripathi S, Golden SS, LiWang A, Partch CL. 2017. Structural basis of the day-night transition in a bacterial circadian clock. Science 355:1174-1180.

24. Maule AF, Wright DP, Weiner JJ, Han L, Peterson FC, Volkman BF, Silvaggi NR, Ulijasz AT. 2015. The aspartate-less receiver (ALR) domains: distribution, structure and function. PLoS Pathog 11:e1004795.

25. Glanville DG, Han L, Maule AF, Woodacre A, Thanki D, Abdullah IT, Morrissey JA, Clarke TB, Yesilkaya H, Silvaggi NR, Ulijasz AT. 2018. RitR is an archetype for a novel family of redox sensors in the streptococci that has evolved from two-component response regulators and is required for pneumococcal colonization. PLoS Pathog 14:e1007052.

26. Su T, Liu S, Wang K, Chi K, Zhu D, Wei T, Huang Y, Guo L, Hu W, Xu S, Lin Z, Gu L. 2015. The REC domain mediated dimerization is critical for FleQ from *Pseudomonas aeruginosa* to function as a c-di-GMP receptor and flagella gene regulator. J Struct Biol 192:1-13.

27. Torres-Sanchez L, Sana TG, Decossas M, Hashem Y, Krasteva PV. 2023. Structures of the *P. aeruginosa* FleQ-FleN master regulators reveal large-scale conformational switching in motility and biofilm control. Proc Natl Acad Sci U S A 120:e2312276120.

28. Chakraborty S, Agarwal S, Bakshi A, Dey S, Biswas M, Ghosh B, Dasgupta J. 2023. The N-terminal FleQ domain of the *Vibrio cholerae* flagellar master regulator FlrA plays pivotal structural roles in stabilizing its active state. FEBS Lett 597:2161-2177.

29. Krasteva PV, Fong JC, Shikuma NJ, Beyhan S, Navarro MV, Yildiz FH, Sondermann H. 2010. *Vibrio cholerae* VpsT regulates matrix production and motility by directly sensing cyclic di-GMP. Science 327:866-8.

30. Wen Y, Ouyang Z, Devreese B, He W, Shao Y, Lu W, Zheng F. 2017. Crystal structure of master biofilm regulator CsgD regulatory domain reveals an atypical receiver domain. Protein Sci 26:2073-2082.

31. Xu Q, Christen B, Chiu HJ, Jaroszewski L, Klock HE, Knuth MW, Miller MD, Elsliger MA, Deacon AM, Godzik A, Lesley SA, Figurski DH, Shapiro L, Wilson IA. 2012. Structure of the pilus assembly protein TadZ from *Eubacterium rectale*: implications for polar localization. Mol Microbiol 83:712-27.

32. Lin W, Wang Y, Han X, Zhang Z, Wang C, Wang J, Yang H, Lu Y, Jiang W, Zhao GP, Zhang P. 2014. Atypical OmpR/PhoB subfamily response regulator GlnR of actinomycetes functions as a homodimer, stabilized by the unphosphorylated conserved Asp-focused charge interactions. J Biol Chem 289:15413-25.

33. Shi J, Feng Z, Xu J, Li F, Zhang Y, Wen A, Wang F, Song Q, Wang L, Cui H, Tong S, Chen P, Zhu Y, Zhao G, Wang S, Feng Y, Lin W. 2023. Structural insights into the transcription activation mechanism of the global regulator GlnR from actinobacteria. Proc Natl Acad Sci U S A 120:e2300282120.

34. Williams SB, Vakonakis I, Golden SS, LiWang AC. 2002. Structure and function from the circadian clock protein KaiA of *Synechococcus elongatus*: a potential clock input mechanism. Proc Natl Acad Sci U S A 99:15357-62.

35. Ye S, Vakonakis I, Ioerger TR, LiWang AC, Sacchettini JC. 2004. Crystal structure of circadian clock protein KaiA from *Synechococcus elongatus*. J Biol Chem 279:20511-8.

36. Pattanayek R, Sidiqi SK, Egli M. 2012. Crystal structure of the redox-active cofactor dibromothymoquinone bound to circadian clock protein KaiA and structural basis for dibromothymoquinone's ability to prevent stimulation of KaiC phosphorylation by KaiA. Biochemistry 51:8050-2.

37. Pattanayek R, Egli M. 2015. Protein-protein interactions in the cyanobacterial circadian clock: Structure of KaiA dimer in complex with C-terminal KaiC peptides at 2.8 A resolution. Biochemistry 54:4575-8.

38. Hickey JM, Lovell S, Battaile KP, Hu L, Middaugh CR, Hefty PS. 2011. The atypical response regulator protein ChxR has structural characteristics and dimer

interface interactions that are unique within the OmpR/PhoB subfamily. J Biol Chem 286:32606-16.

39. O'Hara BP, Norman RA, Wan PT, Roe SM, Barrett TE, Drew RE, Pearl LH. 1999. Crystal structure and induction mechanism of AmiC-AmiR: a ligand-regulated transcription antitermination complex. EMBO J 18:5175-86.

40. Gao R, Bouillet S, Stock AM. 2019. Structural basis of response regulator function. Annu Rev Microbiol 73:175-197.
